# Prevalence of molecular markers of artemisinin and lumefantrine resistance among patients with uncomplicated *Plasmodium falciparum* malaria in three provinces in Angola, 2015

**DOI:** 10.1186/s12936-018-2233-5

**Published:** 2018-02-20

**Authors:** Dragan Ljolje, Pedro Rafael Dimbu, Julia Kelley, Ira Goldman, Douglas Nace, Aleixo Macaia, Eric S. Halsey, Pascal Ringwald, Filomeno Fortes, Venkatachalam Udhayakumar, Eldin Talundzic, Naomi W. Lucchi, Mateusz M. Plucinski

**Affiliations:** 1 Atlanta Research and Education Foundation, Atlanta, GA USA; 2 Malaria Branch, Centers for Disease Control and Prevention, Atlanta, GA USA; 3 National Malaria Control Programme, Ministry of Health, Luanda, Angola; 4 Faculty of Medicine, Agostinho Neto University, Luanda, Angola; 5 U.S. President’s Malaria Initiative, Centers for Disease Control and Prevention, Atlanta, GA USA; 6 Global Malaria Programme, World Health Organization, Geneva, Switzerland

**Keywords:** Malaria, *Plasmodium falciparum*, Angola, Haplotype, *pfmdr1*, *pfk13*, Lumefantrine

## Abstract

**Background:**

Artemisinin-based combination therapy is the first-line anti-malarial treatment for uncomplicated *Plasmodium falciparum* infection in Angola. To date, the prevalence of polymorphisms in the *pfk13* gene, associated with artemisinin resistance, and *pfmdr1*, associated with lumefantrine resistance, have not been systematically studied in Angola.

**Methods:**

DNA was isolated from pretreatment and late treatment failure dried blood spots collected during the 2015 round of therapeutic efficacy studies in Benguela, Lunda Sul, and Zaire Provinces in Angola. The *pfk13* propeller domain and *pfmdr1* gene were sequenced and analysed for polymorphisms. *Pfmdr1* copy number variation was assessed using a real-time PCR method. The association between *pfmdr1* and *pfk13* mutations and treatment failure was investigated.

**Results:**

The majority of pretreatment (99%, 466/469) and all late treatment failure (100%, 50/50) samples were wild type for *pfk13*. Three of the pretreatment samples (1%) carried the A578**S** mutation commonly observed in Africa and not associated with artemisinin resistance. All 543 pretreatment and day of late treatment failure samples successfully analysed for *pfmdr1* copy number variation carried one copy of *pfmdr1.* The NYD haplotype was the predominant *pfmdr1* haplotype, present in 63% (308/491) of pretreatment samples, followed by N**F**D, which was present in 32% (157/491) of pretreatment samples. The *pfmdr1* N86 allele was overrepresented in day of late treatment failure samples from participants receiving artemether–lumefantrine (p value 0.03).

**Conclusions:**

The pretreatment parasites in patients participating in therapeutic efficacy studies in 2015 in Angola’s three sentinel sites showed genetic evidence of susceptibility to artemisinins, consistent with clinical outcome data showing greater than 99% day 3 clearance rates. The lack of increased *pfmdr1* copy number is consistent with previous reports from sub-Saharan Africa. Although *pfmdr1* NYD and N**F**D haplotypes were overrepresented in artemether–lumefantrine late treatment failure samples, their role as markers of resistance was unclear given that these haplotypes were also present in the majority of successfully treated patients in the artemether–lumefantrine treatment arms.

**Electronic supplementary material:**

The online version of this article (10.1186/s12936-018-2233-5) contains supplementary material, which is available to authorized users.

## Background

Artemisinin-based combination therapy (ACT) is among the last effective treatments against *Plasmodium falciparum* malaria in areas where older anti-malarials, such as chloroquine, sulfadoxine–pyrimethamine and mefloquine, have become ineffective due to parasite resistance [1]. To increase clinical efficacy and slow the emergence of parasite resistance, ACT depends on the combined action of two independent drugs. The fast-acting artemisinin derivative, with a half-life on the order of hours, is responsible for the majority of parasite clearing, but is combined with a longer-lasting partner drug with a half-life on the order of days or weeks to clear remaining parasites and result in a lasting clinical cure. Resistance to ACT can involve artemisinin resistance, partner drug resistance, or both [2]. Artemisinin resistance manifests in delayed parasite clearance in the first 3 days following anti-malarial therapy and survival of ring stage parasites [3, 4]. In contrast, partner drug resistance manifests as a late treatment failure, where a patient who initially cleared parasites develops recurrent parasitaemia within 4–6 weeks following anti-malarial therapy.

Artemisinin resistance has been linked to a series of mutations in the *P. falciparum kelch 13* (*pfk13*) propeller domain that prolong parasite clearance time, with C580Y being the best-characterized mutation [5]. Resistance to partner drugs has also been linked to polymorphisms in certain genes: single-nucleotide polymorphisms (SNPs) in *pfcrt* and *pfmdr1* for amodiaquine [6]; increased copy number of the *pfpm2* gene for piperaquine [7, 8]; increased copy number of *pfmdr1* for mefloquine [9, 10]. Increased copy number in *pfmdr1* has been provisionally linked to increased inhibitory concentration for lumefantrine in in vitro studies [10, 11]. Reduced susceptibility to lumefantrine has also been postulated to be associated with both individual *pfmdr1* SNPs including N86 [6, 10, 12–14] as well as certain combinations of SNPs, specifically the N86/184**F**/D1246 haplotype [15, 16].

Angolan national treatment guidelines include three different artemisinin-based combinations for first-line treatment of uncomplicated malaria: artesunate-amodiaquine (ASAQ), dihydroartemisinin–piperaquine (DP), and artemether–lumefantrine (AL). Consistent with World Health Organization (WHO) recommendations, Angola conducts regular therapeutic efficacy monitoring of the three forms of ACT. Data from efficacy studies in Zaire Province in 2013 [17], Zaire Province in 2015 [18] and Luanda Province in 2011–2013 [19] have shown rates of late treatment failure suggestive of lumefantrine resistance in patients treated with AL. Parasites from late treatment failure samples from the 2013 and 2015 Zaire studies were analysed for *pfmdr1* polymorphisms. All AL late treatment failure samples in both years had either the N**F**D or NYD *pfmdr1* haplotype on day of failure. There was no evidence of elevated copy number variation in late treatment failures from 2013; *pfmdr1* copy number was not measured in the 2015 samples. In the Luanda studies, all pretreatment samples were evaluated for *pfmdr1* polymorphisms. The prevalence of the N86 SNP increased from 68% in 2011 to 82% in 2013, and overall 14% of pretreatment samples were found to have an elevated copy number of *pfmdr1*, one of the highest prevalences reported in Africa. However, none of the studies showed either clinical or molecular evidence of artemisinin resistance.

Clinical and molecular data from previous studies underscore the importance of characterizing the parasite population in Angola. Aside from the Luanda efficacy trials that reported data on only 103 participants and a handful of smaller studies [20], there are insufficient data on the population prevalence of key molecular markers of resistance. To gather more data on the prevalence of *pfmdr1* and *pfk13* polymorphisms, pretreatment samples from the 2015 therapeutic efficacy studies in Benguela, Lunda Sul and Zaire Provinces of Angola were genotyped. In addition, the association between these polymorphisms and treatment failure was investigated.

## Methods

A total of 556 dried blood spots collected from children during the 2015 therapeutic efficacy study in Angola from the three provinces of Benguela, Lunda Sul and Zaire were analysed [18]. Of 586 children enrolled and treated with AL, ASAQ or DP, the study had registered 413 cases of adequate clinical and parasitological response (ACPR), 4 cases of early treatment failure, and 50 cases of late treatment failure. The majority of cases of late treatment failure were observed in the AL arm (28), followed by the DP (15) and ASAQ (7) arms. The samples analysed here included 506 pretreatment samples collected at enrollment and the 50 samples collected on any other days when enrolled patients returned with a recurrent malaria infection (day of failure for late treatment failures). All 50 late treatment failure samples had previously been analysed for *pfmdr1* and *pfk13* sequence [18]. DNA was extracted using the QIAamp Blood DNA Kit (Qiagen, Hilden, Germany). The *pfmdr1* and *pfk13* genes were amplified and sequenced using previously described methods [15, 21]. All samples were genotyped for *pfmdr1* copy number variation using a previously described protocol [9]. Geneious R10 software (Biomatters, San Francisco, CA, USA) was used to identify specific SNPs. *Pfmdr1* haplotypes were constructed based on the permutations of SNPs at codons 86, 184, and 1246.

The frequencies of *pfk13* SNPs, *pfmdr1* copy number, *pfmdr1* haplotypes, and *pfmdr1* SNPs in pretreatment samples were calculated stratifying by province. Samples with more than one *pfmdr1* haplotype were included in the numerators for calculations of rates for each *pfmdr1* haplotype present. The association between treatment outcome and either *pfmdr1* or *pfk13* polymorphisms was analysed using two methods. In the first, the prevalence of the polymorphisms in pretreatment samples was compared between patients who experienced ACPR and patients who experienced recrudescence. The relative risk of recrudescence associated with a given polymorphism was calculated as the ratio of the probability of recrudescence in patients carrying the polymorphism prior to treatment versus the probability of recrudescence in patients with wild type pretreatment parasites. In the second method, the prevalence of polymorphisms was compared between pretreatment samples from cases of ACPR and day of failure samples from all cases of late treatment failure (recrudescence and reinfection). In both methods, statistical significance of the association was tested using a Fisher’s exact test.

Parents or guardians provided written permission upon enrollment. The study was approved by human subjects research boards at the Angolan Ministry of Health and the WHO and was approved as a non-research surveillance activity by the Office of the Associate Director for Science in the Center for Global Health at the Centers for Disease Control and Prevention (CDC) (Protocol 2014-233).

## Results

For the pretreatment samples, sequences of the *pfk13* gene were obtained for 469/469 (100%), *pfmdr1* copy number was measured in 497/506 (98%), and sequences of the *pfmdr1* gene were obtained for 491/506 (96%) (Table 1). For the day of late treatment failure samples, sequences of the *pfk13* gene were obtained for 50/50 (100%), *pfmdr1* copy number was measured for 46/49 (94%), and sequences of the *pfmdr1* gene were obtained for 50/50 (100%).

**Table 1:** Total number of samples successfully amplified for *pfmdr1* copy number and sequenced for *pfmdr1* and *pfk13* from therapeutic efficacy monitoring in Angola, 2015

	Benguela	Lunda Sul	Zaire	Total
Pretreatment				
*pfk13* sequence	188/188 (100%)	82/82 (100%)	199/199 (100%)	469/469 (100%)
*pfmdr1* copy number	195/199 (98%)	99/101 (98%)	203/206 (99%)	497/506 (98%)
*pfmdr1* sequence	194/198 (97%)	96/101 (95%)	201/207 (97%)	491/506 (96%)
Day of late treatment failure				
*pfk13* sequence	15/15 (100%)	0	35/35 (100%)	50/50 (100%)
*pfmdr1* copy number	15/15 (100%)	0	31/34 (91%)	46/49 (94%)
*pfmdr1* sequence	15/15 (100%)	0	35/35 (100%)	50/50 (100%)
Total samples				
*pfk13* sequence	203/203 (100%)	82/82 (100%)	234/234 (100%)	519/519 (100%)
*pfmdr1* copy number	210/214 (98%)	99/101 (98%)	234/240 (98%)	543/555 (98%)
*pfmdr1* sequence	209/213 (97%)	96/101 (95%)	236/242 (97%)	541/556 (97%)

Three of 469 pretreatment samples (1%) had the A578**S** *pfk13* mutation, and the remaining samples were wild type for *pfk13.* Two of the A578**S** *pfk13* mutants were from Lunda Sul, and one was from Zaire. An additional sample from Zaire carried a synonymous mutation T535T (Table 2). All day of late treatment failure samples were wild type for *pfk13*, but two recrudescent infections from Zaire carried one synonymous mutation each: T535T, which was also present in the participant’s pretreatment sample, and P553P, which was not present at enrollment. All 543 combined pretreatment and day of late treatment failure samples where *pfmdr1* copy number was measured had only one copy.

**Table 2:** Prevalence of *pfk13* and *pfmdr1* polymorphisms from pretreatment and day of late treatment failure observed during therapeutic efficacy monitoring in Angola, 2015

Genetic markers	Pretreatment				Day of late treatment failure			
	Benguela	Lunda Sul	Zaire	Total	Benguela	Lunda Sul	Zaire	Total
*pfk13*	n = 188	n = 82	n = 199	n = 469	n = 15	n = 0	n = 35	n = 50
Wild type	188 (100%)	80 (98%)	198 (99%)^b^	466 (99%)	15 (100%)	0	35 (100%)^c^	50 (100%)
A578**S**	0	2 (2%)	1 (< 1%)	(< 1%)	0	0	0	0
*pfmdr1* copy number	n = 195	n = 99	n = 203	n = 497	n = 15	0	n = 31	n = 46
1	195 (100%)	99 (100%)	203 (100%)	497 (100%)	15 (100%)	0	31 (100%)	46 (100%)
*pfmdr1* haplotypes^a^	n = 194	n = 96	n = 201	n = 491	n = 15	0	n = 35	n = 50
NYD	133 (69%)	68 (71%)	106 (54%)	308 (63%)	12 (80%)	0	21 (60%)	33 (66%)
**Y**YD	25 (13%)	6 (6%)	31 (16%)	62 (13%)	0	0	1 (3%)	1 (2%)
N**F**D	49 (25%)	34 (35%)	74 (37%)	157 (32%)	5 (33%)	0	13 (37%)	18 (36%)
**YF**D	1 (< 1%)	1 (1%)	13 (7%)	15 (3%)	0	0	1 (3%)	1 (2%)
**Y**Y**Y**	0	0	6 (3%)	6 (1%)	1 (7%)	0	0	1 (2%)
NY**Y**	0	0	1 (< 1%)	1 (< 1%)	0	0	0	0
**YFY**	0	0	1 (< 1%)	1 (< 1%)	0	0	0	0

^a^Participants with mixed infections were included in the numerator for each haplotype 86, 184, 1246 observed; *pfmdr1* haplotypes are N86Y, Y184F, D1246Y

^b^Includes one sample with synonymous mutation T535T

^c^Includes one sample with synonymous mutation T535T and one with synonymous mutation P553P

The NYD haplotype (N86, Y184, D1246) was the predominant *pfmdr1* haplotype, present in 308/491 (63%) of all pretreatment samples, ranging from 106/201 (54%) in Zaire to 133/194 (69%) in Benguela, and 68/96 (71%) in Lunda Sul (Table 2). The N**F**D haplotype (N86, 184F, D1246) was the second most prevalent haplotype, present in 157/491 (32%) of all pretreatment samples, ranging from 49/194 (25%) in Benguela, 34/96 (34%) in Lunda Sul, and 74/201 (37%) in Zaire. Haplotype **Y**YD (86Y, Y184, D1246) was present in 62/491 (13%) of all pretreatment samples, ranging from 25/194 (13%) in Benguela, 31/201 (16%) in Zaire and 6/96 (6%) in Lunda Sul. Other haplotypes were present at lower frequencies: **YF**D 15/491 (3%), **Y**Y**Y** 6/491 (1%), NY**Y** 1/491 (< 1%) and **YFY** 1/491 (< 1%).

For day of late treatment failure samples, haplotype NYD was identified in 12/15 (80%) for Benguela and 21/35 (60%) in Zaire for an aggregate of 33/50 (66%). Haplotype N**F**D was identified in 18/50 (36%) of day of late treatment failure samples, ranging from 5/15 (33%) in Benguela to 13/35 (37%) in Zaire.

Participants infected with parasites carrying the **Y**YD haplotype had a lower risk of AL recrudescence (relative risk ratio of 0.3) compared to those with an infection with parasites having the NYD haplotype at enrollment, but this was not statistically significant (p value 0.4) (Table 3; see Additional file 1). However, after accounting for recrudescence plus reinfections, the NYD haplotype was significantly more likely to be found in late treatment failure samples than the **Y**YD haplotype (p value 0.04). The *pfmdr1* N86 SNP was overrepresented in recrudescence plus reinfection samples from participants treated with AL (p value 0.03). No other *pfmdr1* haplotype or SNP was associated with AL treatment response. There were no statistically significant associations between *pfmdr1* haplotypes or SNPs and DP or ASAQ treatment response.

**Table 3:** Association between *pfk13* and *pfmdr1* polymorphisms and late treatment failures observed during therapeutic efficacy studies in Angola, 2015, stratified by treatment arm

Genetic markers	Artemether–lumefantrine (28 late treatment failures)			Dihydroartemisinin–piperaquine (15 late treatment failures)			Artesunate–amodiaquine (7 late treatment failures)		
	RR^†^	P value^††^	P value^†††^	RR^†^	P value^††^	P value^†††^	RR^†^	P value^††^	P value^†††^
*pfk13*									
Wild type	Ref	Ref	–	Ref	Ref	–	–	–	–
A578**S**	0	1	–	0	1	–	–	–	–
*pfmdr*1 copy number									
1	–	–	–	–	–	–	–	–	–
*pfmdr*1 haplotypes									
NYD	Ref	Ref	Ref	Ref	Ref	Ref	Ref	Ref	Ref
**Y**YD	0.3	0.4	*0.04*	0	1	0.7	0	1	0.6
N**F**D	1.0	1	0.7	1.75	1	1	0	1	1
**YF**D	2.0	0.4	0.6	0	1	0.5	–	–	–
**Y**Y**Y**	0	1	0.6	0	1	1	–	–	0.1
NY**Y**	0	1	1	–	–	–	–	–	–
**YFY**	0	1	1	–	–	–	–	–	–
*pfmdr1* single nucleotide polymorphisms									
N86	Ref	Ref	Ref	Ref	Ref	Ref	Ref	Ref	Ref
86**Y**	0.3	0.5	*0.03*	0	1	0.5	0	1	0.1
Y184	Ref	Ref	Ref	Ref	Ref	Ref	Ref	Ref	Ref
184**F**	1.3	0.7	0.3	0	1	0.8	0	1	1
D1246	Ref	Ref	Ref	Ref	Ref	Ref	Ref	Ref	Ref
1246**Y**	0	1	0.6	0	1	1	–	–	0.07

^†^Relative risk (RR) of treatment failure (recrudescence)

^††^Statistical significance of difference in risk of treatment failure (recrudescence)

^†††^Statistical significance of difference in risk of treatment failure (recrudescence or reinfection)

## Discussion

With the ongoing challenges related to artemisinin resistance in Southeast Asia and threat of resistance in Africa, monitoring treatment efficacy and genetic markers associated with anti-malarial drug resistance in Angola and the rest of malaria-endemic Africa remains imperative. While pretreatment samples from therapeutic efficacy studies are not necessarily representative of the true population prevalence of these markers due to the limited temporal and geographical sampling inherent to therapeutic efficacy monitoring, they can provide more information on the population prevalence of these markers than analysis of the treatment failures alone.

The findings reported in this study are consistent with those from reports from other geographic regions where malaria parasites remain highly sensitive to artemisinin derivatives. No *pfk13* mutations associated with artemisinin resistance were found, and the only *pfk13* polymorphism detected was the A578**S** mutation, which has been observed throughout Africa [22]. All three participants with parasites carrying this mutation were successfully treated, consistent with other studies that have not found an association between this mutation and artemisinin resistance [22]. The observation of synonymous mutations on the day of recrudescence in two patients is notable, but it is not possible to associate these with artemisinin resistance.

The findings reported here that there was only a single copy of *pfmdr1* in all 543 tested samples from 2015, the largest analysis of *pfmdr1* copy number to date in Angola, correspond with the findings reported from the 2013 efficacy monitoring in Zaire and Uíge Provinces, but are not consistent with the previous report of a 14% prevalence of elevated *pfmdr1* copy number in 2011–2013 in Luanda [19]. Luanda, a large city with major international connections, is more than 500 km away by road from Zaire, Benguela, and Lunda Sul, so this discrepancy could reflect geographic heterogeneity in *pfmdr1* copy number variation. Similarly, a 2–4 year difference in time of sample collection could mean that separate parasite populations with different rates of *pfmdr1* copy number were circulating during the different study periods or selected for at the different facilities where the studies were conducted. Alternately, these differences could result from differences in how the copy number variation was assessed. The Luanda study performed a previously described real-time PCR using SYBR Green I dye for the detection of *pfmdr1* copy number [23], whereas the analysis reported here used a TaqMan probe-based real-time PCR method [9].

The majority of pretreatment samples carried the N86 allele in *pfmdr1*, an important cautionary finding given previous studies demonstrating significantly reduced susceptibility of *P. falciparum* to lumefantrine in parasites harboring the N86 allele when compared to the N86**Y** allele [6, 12]. In line with these findings, in the present study N86 was also found to be over-represented in day of late treatment failure samples compared to pretreatment samples in patients successfully treated with AL. However, 75% of patients who responded adequately following treatment with AL also had pretreatment parasites with the N86 allele; therefore, the predictive value of this codon alone as a marker for AL treatment failure is limited. No statistically significant associations between *pfk13* and *pfmdr1* polymorphisms and treatment outcome were observed in any of the non-AL arms; however the number of treatment failures in the ASAQ and DP arms was low.

Ultimately, therapeutic efficacy studies are not powered to test for the association between parasite genotypes and treatment outcome. Assuming a 5% treatment failure rate in wild type parasites and a 10% prevalence of a mutant genotype, a single therapeutic efficacy study would need to enroll 2430 participants to have 80% power to detect a doubling in the risk of treatment failure in participants with the mutant allele. Pooling results across multiple therapeutic efficacy studies is one way of using therapeutic efficacy data to identify molecular markers of treatment failure over time, and can also allow inclusion of other potential covariates such as patient age, drug dose, and baseline parasitemia. Therapeutic efficacy investigators are encouraged to make available any genotyping data on pretreatment and day of late treatment failure samples.

## Additional file

**Additional file 1.** Association between *pfk13* and *pfmdr1* polymorphisms and late treatment failures observed during therapeutic efficacy studies in Angola, 2015, stratified by treatment, full data.
