# Supplementary material for: Prevalence of molecular markers of artemisinin and lumefantrine resistance among patients with uncomplicated Plasmodium falciparum malaria in three provinces in Angola, 2015
Source: Malar J. 2018 Feb 20;17:84. doi: 10.1186/s12936-018-2233-5 (PMC5819161; doi:10.1186/s12936-018-2233-5)
Supplement: Supplementary file 1 — Additional file 1. Association between pfk13 and pfmdr1 polymorphisms and late treatment failures observed during therapeutic efficacy studies in Angola, 2015, stratified by treatment, full data. [file 12936_2018_2233_MOESM1_ESM.pdf]

Additional File 1: Association between *pfk13* and *pfmdr1* polymorphisms and late treatment failures observed during therapeutic efficacy studies in Angola, 2015, stratified by treatment, full data

Artemether-lumefantrine

	ACPR	Recrudescence (DF)	RR†	P-value††	Recrudescence + Reinfection (DF)	P-value†††
pkf13						
Wildtype	165/166 (99%)	9/9 (100%)	-	-	28/28 (100%)	-
A578S	1/166 (<1%)	0	0	**1**	-	-
***pfmdr1* copy number**						
1	171/171 (100%)	9/9 (100%)	-	-	26/26 (100%)	-
***pfmdr1* haplotype**						
**NYD**	**92/161 (57%)**	**8/9 (89%)**	**Ref**	**Ref**	**23/28 (82%)**	**Ref**
YYD	35/161 (22%)	1/9 (11%)	0.3	0.4	2/28 (7%)	0.04
NFD	44/161 (27%)	4/9 (44%)	1.0	1	13/28 (46%)	0.7
YFD	5/161 (3%)	1/9 (11%)	2.0	0.4	2/28 (7%)	0.6
YYY	5/161 (3%)	0	0	1	0	0.6
NYY	0	0	0	1	0	1
YFY	1/161 (<1%)	0	0	1	0	1
***pfmdr1* SNP**						
**N86**	**121/161(75%)**	**9/9 (100%)**	**Ref**	**Ref**	**27/28 (96%)**	**Ref**
86Y	42/161 (26%)	1/9 (11%)	0.3	0.5	2/28 (7%)	**0.03**
**Y184**	**128/161 (80%)**	**8/9 (89%)**	**Ref**	**Ref**	**23/28 (82%)**	**Ref**
184F	48/161 (30%)	4/9 (44%)	1.3	0.7	13/28 (46%)	0.3
**D1246**	**155/161 (96%)**	**9/9 (100%)**	**Ref**	**Ref**	**27/28 (96%)**	**Ref**
1246Y	6/161 (4%)	0	0	1	0	0.6

Dihydroartemisinin-piperaquine

	ACPR	Recrudescence (DF)	RR†	P-value††	Recrudescence + Reinfection (DF)	P-value†††
pkf13						
Wildtype	165/167 (99%)	1/1 (100%)	-	-	15/15 (100%)	-
A578S	2/167 (1%)	0	0	**1**	-	-
***pfmdr1* copy number**						
1	185/185 (100%)	0	-	-	13/13 (100%)	-
***pfmdr1* haplotype**						
**NYD**	**116/182 (64%)**	**1/1 (100%)**	**Ref**	**Ref**	**12/15 (80%)**	**Ref**
YYD	15/182 (8%)	0	0	1	2/15 (13%)	0.7
NFD	66/182 (36%)	1/1 (100%)	1.75	1	7/15 (47%)	1
YFD	6/182 (3%)	0	0	1	1/15 (7%)	0.5
YYY	1/182 (<1%)	0	0	1	0	1
NYY	0	0	-	-	0	-
YFY	0	0	-	-	0	-
***pfmdr1* SNP**						
**N86**	**163/182 (90%)**	**1/1 (100%)**	**Ref**	**Ref**	**15/15 (100%)**	**Ref**
86Y	22/182 (12%)	0	0	1	3/15 (20%)	0.5
**Y184**	**129/182 (71%)**	**1/1 (100%)**	**Ref**	**Ref**	**13/15 (86%)**	**Ref**
184F	71/182 (39%)	0	0	1	8/15 (53%)	0.8
**D1246**	**181/182 (99%)**	**1/1 (100%)**	**Ref**	**Ref**	**15/15 (100%)**	**Ref**
1246Y	1/182(<1%)	0	0	1	0	1

Artesunate-amodiaquine

	ACPR	Recrudescence (DF)	RR†	P-value††	Recrudescence + Reinfection (DF)	P-value
pkf13						
Wildtype	94/94 (100%)	0	-	-	7/7(100%)	-
A578S						
***pfmdr1* copy number**						
1	90/90 (100%)	0	-	-	7/7 (100%)	-
***pfmdr1* haplotype**						
**NYD**	**62/92 (67%)**	**0**	**Ref**	**Ref**	**6/7 (86%)**	**Ref**
YYD	7/92 (8%)	0	0	1	1/7 (17%)	0.6
NFD	28/92 (30%)	0	0	1	2/7 (29%)	1
YFD	0	0	-	-	0	-
YYY	0	0	-	-	1/7 (14%)	0.1
NYY	0	0	-	-	0	-
YFY	0	0	-	-	0	-
***pfmdr1* SNP**						
**N86**	**86/92 (93%)**	**0**	**Ref**	**Ref**	**6/7 (86%)**	**Ref**
86Y	7/92 (8%)	0	0	1	2/7 (29%)	0.1
**Y184**	**68/92 (74%)**	**0**	**Ref**	**Ref**	**7/7 (100%)**	**Ref**
184F	28/92 (30%)	0	0	1	2/7 (29%)	1
**D1246**	**92/92 (100%)**	**0**	**Ref**	**Ref**	**6/7 (86%)**	**Ref**
1246Y	0	0	-	-	1/7 (14%)	0.07

SNP: single nucleotide polymorphism; ACPR: adequate clinical and parasitological failure; DF: day of failure; RR: relative risk; Ref: reference

† Relative risk of treatment failure (recrudescence)

†† Statistical significance of difference in risk of treatment failure (recrudescence)

††† Statistical significance of difference in risk of treatment failure (recrudescence or reinfection)

†††
